# Extraction strategy influences the phytochemical composition and multifunctional biological activities of *Genista albida*: implications for functional food development

**DOI:** 10.1039/d6ra06022f

**Published:** 2026-08-10

**Authors:** Cengiz Sarikurkcu, Aysegul Dinc, Ilkay Kir

**Affiliations:** a Faculty of Pharmacy, Afyonkarahisar Health Sciences University TR-03100 Afyonkarahisar Türkiye sarikurkcu@gmail.com +90-507 191 25 24

## Abstract

*Genista albida* Willd., an endemic species of Türkiye, represents a largely unexplored source of bioactive phytochemicals with potential applications in functional foods. This study investigated how extraction strategy influences the phytochemical composition and biological activities of *G. albida* by comparing ultrasonic-assisted extraction (UAE), maceration (MAC), and Soxhlet extraction (SOE). Total phenolic and flavonoid contents were determined spectrophotometrically, while phenolic constituents were characterized by LC-MS/MS. Antioxidant capacity was evaluated using six complementary assays, and enzyme inhibitory activities against acetylcholinesterase, butyrylcholinesterase, tyrosinase, α-amylase, and α-glucosidase were assessed. Twenty-one of the 31 targeted phenolic compounds were identified, with hesperidin as the predominant constituent (4378–4584 µg per g extract), followed by hyperoside, luteolin 7-*O*-glucoside, ferulic acid, eriodictyol, apigenin, and luteolin. UAE yielded the highest total phenolic content (43.28 mg GAE per g extract) and the strongest overall antioxidant performance, whereas MAC exhibited the highest flavonoid content (50.59 mg RE per g extract) and acetylcholinesterase inhibitory activity. Notably, all extracts demonstrated stronger α-glucosidase inhibition than acarbose (IC_50_ = 0.63–0.66 *vs.* 0.96 mg mL^−1^), highlighting their potential to modulate postprandial hyperglycemia. Overall, extraction strategy markedly affected phytochemical recovery and bioactivity, with UAE providing the most favorable balance between chemical richness and biological performance. These findings position *G. albida* as a promising natural source of bioactive ingredients for the development of functional foods and nutraceuticals.

## Abstract 2

Extraction strategy influences the phytochemical profile and biological activities of *Genista albida*. LC-MS/MS highlights its potential for functional food and nutraceutical applications.

## Graphical abstract

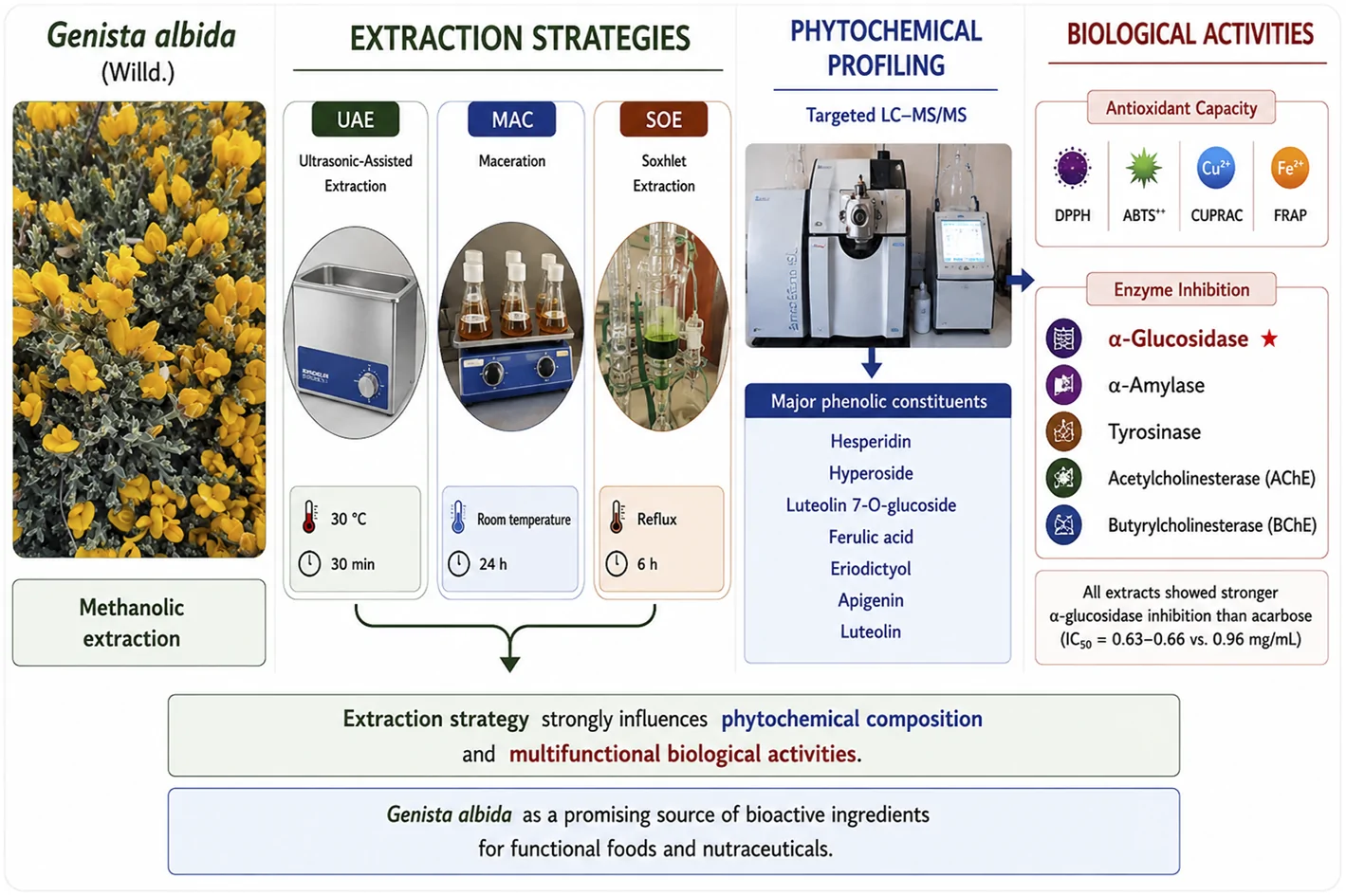

## Introduction

Plant-derived bioactive compounds have attracted considerable attention as functional food ingredients owing to their ability to promote health beyond basic nutrition. In particular, phenolic compounds are recognized for their multifunctional biological properties, including antioxidant, anti-inflammatory, neuroprotective, antimicrobial, and antidiabetic activities, making them valuable candidates for the development of functional foods and nutraceuticals.^1,2^ Among dietary strategies for managing type 2 diabetes mellitus, inhibition of carbohydrate-hydrolyzing enzymes, particularly α-glucosidase, has emerged as an effective approach for controlling postprandial hyperglycemia. By delaying the hydrolysis of dietary carbohydrates and subsequent intestinal glucose absorption, α-glucosidase inhibitors effectively reduce postprandial glucose excursions. Consequently, increasing attention has been directed toward identifying natural α-glucosidase inhibitors from plant sources as safer alternatives or complementary agents to conventional therapeutic approaches.^1,3^

Among plant secondary metabolites, flavonoids represent one of the most extensively investigated phytochemical classes because of their remarkable structural diversity and broad spectrum of biological activities. Numerous flavonoids, including flavanones and flavones, have been reported to exhibit potent antioxidant properties while simultaneously modulating metabolically important enzymes such as α-glucosidase, α-amylase, cholinesterases, and tyrosinase, highlighting their potential as multifunctional dietary bioactives.^1,3^ However, the biological performance of plant extracts depends not only on their phytochemical composition but also on the efficiency with which these compounds are recovered during extraction, emphasizing the importance of selecting appropriate extraction strategies for maximizing their functional value.

Species belonging to the genus *Genista* (Fabaceae) have gained increasing scientific interest because of their rich phytochemical composition and diverse biological properties. Distributed mainly throughout the Mediterranean region, Europe, and Western Asia, *Genista* species are recognized as important sources of flavonoids, isoflavonoids, alkaloids, and phenolic acids that contribute to antioxidant, anti-inflammatory, antimicrobial, neuroprotective, and antidiabetic activities.^1,2^ Recent phytochemical investigations have identified hesperidin, hyperoside, luteolin derivatives, apigenin derivatives, and related phenolics as characteristic constituents of several *Genista* species, many of which have been associated with pronounced antioxidant and enzyme inhibitory activities.^2–4^ Beyond their antioxidant properties, increasing evidence suggests that *Genista* species may represent promising natural sources of α-glucosidase inhibitors, highlighting their potential application in functional foods designed for glycemic management. Nevertheless, considerable interspecific variation exists in phytochemical composition and biological activity, indicating that the functional potential of each species should be evaluated individually.^1,3^

Extraction strategy is one of the principal factors determining the recovery, composition, and biological performance of plant-derived phytochemicals. Although conventional techniques such as maceration and Soxhlet extraction remain widely employed, emerging extraction technologies including ultrasonic-assisted extraction (UAE) have attracted considerable interest because they may selectively recover different phytochemical classes while improving extraction efficiency and reducing processing time and solvent consumption.^5–7^ Importantly, no single extraction method is universally optimal, as extraction selectivity depends on the physicochemical characteristics of target metabolites. Consequently, differences in extraction strategy may substantially influence not only phytochemical composition but also antioxidant capacity and enzyme inhibitory activities. Comparative evaluation of extraction methods is therefore essential for identifying extraction conditions that maximize the recovery of biologically active constituents with potential applications in functional foods and nutraceuticals.^5–7^

*Genista albida* Willd. is an endemic species of Türkiye whose phytochemical composition and biological potential remain largely unexplored despite the growing interest in *Genista*-derived bioactive compounds.^4^

Although several *Genista* species have been phytochemically characterized, evidence regarding the influence of extraction strategy on the recovery of bioactive constituents and their associated biological activities in *G. albida* remains unavailable. Therefore, the present study comparatively evaluated methanolic extracts of *G. albida* obtained by ultrasonic-assisted extraction, maceration, and Soxhlet extraction through comprehensive phytochemical characterization, including extraction yield, total phenolic and flavonoid contents, and LC-MS/MS-based targeted profiling, followed by the evaluation of antioxidant capacity and inhibitory activities against acetylcholinesterase, butyrylcholinesterase, tyrosinase, α-amylase, and α-glucosidase. To the best of our knowledge, this study represents the first comprehensive investigation comparing these extraction techniques for *G. albida* under identical solvent conditions while systematically integrating phytochemical profiling, multiple antioxidant assays, enzyme inhibitory activities, and multivariate correlation analysis. This integrated approach enables a comprehensive evaluation of how extraction strategy influences both the qualitative and quantitative composition of phenolic compounds and their associated multifunctional biological activities. By elucidating the relationship between extraction strategy, phytochemical composition, biological activities, and α-glucosidase inhibitory activity, this study expands the current phytochemical knowledge of the endemic species *G. albida* and provides a scientific basis for selecting appropriate extraction strategies for the development of phytochemical-rich functional food ingredients and nutraceuticals.

## Materials and methods

### Plant material

Aerial parts of *Genista albida* Willd. (Fabaceae) were collected during the flowering period in June 2025 from natural populations in the vicinity of Sertavul Pass, located between Mersin and Karaman provinces in the Anatolian region of Türkiye. The botanical identification of the plant material was performed by Prof. Dr Muhittin Dinç from the Ahmet Keleşoğlu Faculty of Education, Necmettin Erbakan University, and a voucher specimen was deposited in the herbarium under the accession number M. Dinç 3689 for future reference. The collected aerial parts (stems, leaves, and flowers) were thoroughly cleaned to remove soil and debris, then air-dried in the shade at room temperature (20–25 °C) for approximately 10–14 days until constant weight was achieved. The dried plant material was ground into a fine powder using a mechanical grinder and passed through a 40-mesh sieve to ensure particle size uniformity. The powdered material was stored in airtight amber glass containers at room temperature in a dark, dry place until extraction.

### Extraction procedures

Three different extraction methods were employed to prepare methanolic extracts from *G. albida* aerial parts, using methanol as the extraction solvent for all procedures. Methanol was selected because it is widely used in analytical phytochemical investigations owing to its high extraction efficiency for a broad spectrum of phenolic compounds, favorable polarity, and excellent compatibility with LC-MS/MS analysis.^5,6,8^ Moreover, the primary objective of the present study was to compare the performance of different extraction strategies under identical solvent conditions while minimizing solvent-related variability. Therefore, the same solvent was used throughout all extraction procedures to ensure that the observed differences were attributable to the extraction techniques rather than to solvent effects. Although methanol is not considered a food-grade solvent, the present work was designed as an analytical phytochemical screening and comparative extraction study. Future studies should investigate food-grade solvents, particularly ethanol and hydroethanolic mixtures, to facilitate the translation of these findings into functional food and nutraceutical applications.^6,8^

### Ultrasonic-assisted extraction (UAE)

Powdered aerial parts (10 g) were extracted with 100 mL methanol (1 : 10, w/v) using an ultrasonic bath (40 kHz, 300 W, 30 °C) for 30 min. The extraction was repeated twice, and combined filtrates were concentrated under reduced pressure at 40 °C using a rotary evaporator. Extracts were weighed, extraction yields were calculated, and samples were stored at −20 °C until analysis.

### Maceration (MAC)

Powdered aerial parts of *G. albida* (10 g) were macerated with 100 mL of methanol (1 : 10, w/v) in an amber glass bottle at room temperature (20–25 °C) for 24 h under continuous stirring (150 rpm). The extract was filtered through Whatman No. 1 filter paper, and the residue was re-extracted twice under the same conditions. The combined filtrates were concentrated under reduced pressure at 40 °C using a rotary evaporator (Heidolph Laborota 4000, Germany). The crude extract was weighed to determine extraction yield and stored at −20 °C in amber glass vials until further analysis.

### Soxhlet extraction (SOE)

Powdered aerial parts of *G. albida* (10 g) were extracted with 150 mL of methanol using a Soxhlet apparatus for 6 h. After extraction, the solvent was removed under reduced pressure at 40 °C using a rotary evaporator (Heidolph Laborota 4000, Germany). The crude extract was weighed to determine extraction yield and stored at −20 °C in amber glass vials until further analysis.

### Determination of total phenolic content (TPC)

Total phenolic content (TPC) was determined using the Folin–Ciocalteu colorimetric method according to^9^ with slight modifications.^10,11^ Briefly, 0.5 mL of extract solution was mixed with 2.5 mL of Folin–Ciocalteu reagent (1 : 10, v/v, diluted with distilled water). After 5 min, 2.0 mL of sodium carbonate solution (7.5%, w/v) was added, and the mixture was incubated at room temperature in the dark for 90 min. Absorbance was measured at 760 nm using a UV–Vis spectrophotometer (Shimadzu UV-1800, Japan). Gallic acid (10–100 µg mL^−1^) was used to construct the calibration curve, and the results were expressed as milligrams of gallic acid equivalents per gram of dry extract (mg GAE per g extract). All analyses were performed in triplicate, and the results are presented as mean ± standard deviation.

### Determination of total flavonoid content (TFC)

Total flavonoid content (TFC) was determined using the aluminum chloride (AlCl_3_) colorimetric method according to^12^ with slight modifications.^13–15^ Briefly, 1.0 mL of extract solution was mixed with 1.0 mL of aluminum chloride solution (2%, w/v in methanol). A corresponding blank was prepared by replacing the aluminum chloride solution with methanol. After incubation at room temperature for 10 min, absorbance was measured at 415 nm using a UV–Vis spectrophotometer (Shimadzu UV-1800, Japan). Rutin (10–100 µg mL^−1^) was used for calibration, and the results were expressed as milligrams of rutin equivalents per gram of dry extract (mg RE per g extract). All analyses were performed in triplicate, and the results are presented as mean ± standard deviation.

### LC-MS/MS analysis

Phenolic compounds were identified and quantified by LC–ESI–MS/MS according to the validated method of.^16^ Analyses were performed using an Agilent 1260 Infinity liquid chromatography system coupled to an Agilent 6420 Triple Quad mass spectrometer (Agilent Technologies, Santa Clara, CA, USA). Chromatographic separation was achieved on a Poroshell 120 EC-C18 column (100 × 4.6 mm, 2.7 µm) maintained at 25 °C. The mobile phase consisted of solvent A (0.1%, v/v formic acid in water) and solvent B (methanol), using the following gradient program: 0–3 min, 2% B; 6 min, 25% B; 10 min, 50% B; 14–17 min, 95% B; and 17.5 min, 2% B. The flow rate was 0.4 mL min^−1^, and the injection volume was 2 µL.

Mass spectrometric detection was performed using an electrospray ionization (ESI) source operated in both positive and negative multiple reaction monitoring (MRM) modes, depending on the analyte. The optimized source parameters were as follows: capillary voltage, −3.5 kV; gas temperature, 300 °C; gas flow, 11 L min^−1^; and nebulizer pressure, 40 psi. Compound identification and quantification were based on retention times and optimized MRM transitions of authentic standards. A total of 31 phenolic compounds were targeted for analysis, and the corresponding MRM transitions and analytical parameters are provided in SI Table S1.

### Antioxidant activity assays

The antioxidant capacities of *G. albida* extracts were evaluated using six complementary *in vitro* assays representing different mechanisms of antioxidant action: phosphomolybdenum total antioxidant capacity,^17^ DPPH radical scavenging,^18^ ABTS radical scavenging,^19^ CUPRAC reducing power,^20^ FRAP reducing power,^21^ and ferrous ion chelating activity,^22^ with slight modifications.^13,23–25^

For all assays, Trolox (6-hydroxy-2,5,7,8-tetramethylchroman-2-carboxylic acid) was used as the positive control for DPPH, ABTS, CUPRAC, FRAP, and phosphomolybdenum assays, while EDTA (ethylenediaminetetraacetic acid) was used as the positive control for the metal chelating assay. All measurements were performed in triplicate.

### Phosphomolybdenum assay

This assay measures the total antioxidant capacity based on the reduction of Mo(vi) to Mo(v) by the extract, forming a green phosphate/Mo(v) complex at acidic pH. Extract solutions at various concentrations were mixed with phosphomolybdenum reagent (0.6 M sulfuric acid, 28 mM sodium phosphate, and 4 mM ammonium molybdate) and incubated at 95 °C for 90 minutes. After cooling, absorbance was measured at 695 nm. Results were expressed as EC_50_ values (concentration producing an absorbance of 0.500) and as Trolox equivalents.

### CUPRAC (cupric ion reducing antioxidant capacity) assay

This assay measures the reducing capacity of antioxidants based on the reduction of Cu(ii) to Cu(i) by antioxidant compounds. Extract solutions were mixed with copper(ii) chloride solution, neocuproine alcoholic solution, and ammonium acetate buffer (pH 7.0), then incubated at room temperature for 30 minutes. Absorbance was measured at 450 nm. Results were expressed as EC_50_ values and Trolox equivalents.

### FRAP (ferric reducing antioxidant power) assay

This assay measures the ability of antioxidants to reduce Fe(iii)-TPTZ complex to the blue-colored Fe(ii)-TPTZ complex. Extract solutions were mixed with freshly prepared FRAP reagent (acetate buffer pH 3.6, TPTZ solution in HCl, and ferric chloride solution in ratio 10 : 1:1) and incubated at 37 °C for 30 minutes. Absorbance was measured at 593 nm. Results were expressed as EC_50_ values and Trolox equivalents.

### DPPH (2,2-diphenyl-1-picrylhydrazyl) radical scavenging assay

This assay measures the ability of antioxidants to scavenge the stable DPPH free radical. Extract solutions at various concentrations were mixed with DPPH methanolic solution (0.1 mM) and incubated in the dark for 30 minutes. Absorbance was measured at 517 nm. Percent inhibition was calculated, and results were expressed as IC_50_ values (concentration required for 50% inhibition) and Trolox equivalents.

### ABTS (2,2′-azinobis-(3-ethylbenzothiazoline-6-sulfonic acid)) radical cation scavenging assay

This assay measures the scavenging of the blue-green ABTS radical cation (ABTS˙^+^). The ABTS radical cation was generated by reacting ABTS stock solution with potassium persulfate and allowing the mixture to stand in the dark for 12–16 hours. Extract solutions were mixed with diluted ABTS˙^+^ solution and incubated for 6 minutes. Absorbance was measured at 734 nm. Results were expressed as IC_50_ values and Trolox equivalents.

### Metal chelating assay

This assay measures the ability of extracts to chelate ferrous ions (Fe^2+^), thereby preventing the formation of the Fe^2+^-ferrozine complex. Extract solutions were mixed with ferrous chloride solution and ferrozine reagent, then incubated for 10 minutes at room temperature. Absorbance was measured at 562 nm. Percent chelating activity was calculated, and results were expressed as IC_50_ values and EDTA equivalents.

### Relative Antioxidant Capacity Index (RACI)

Prior to relative antioxidant capacity index calculation, antioxidant assay results expressed as IC_50_ or EC_50_ values were adjusted so that lower values corresponded to higher antioxidant capacity, ensuring consistency in data standardization. The RACI was calculated to enable an integrated comparison of antioxidant activities obtained from different assays, as previously described.^26,27^ RACI values were obtained by standardizing individual antioxidant assay results based on their mean and standard deviation and then averaging the standardized scores for each extract.

### Enzyme inhibition assays

Enzyme inhibition assays were performed using acetylcholinesterase from *Electrophorus electricus* (electric eel), butyrylcholinesterase from equine serum, tyrosinase from *Agaricus bisporus* (mushroom), α-amylase from porcine pancreas, and α-glucosidase from *Saccharomyces cerevisiae*. All enzymes were purchased from Sigma-Aldrich (St. Louis, MO, USA).

The enzyme inhibitory activities of *G. albida* extracts were evaluated using well-established spectrophotometric assays: acetylcholinesterase (AChE) and butyrylcholinesterase (BChE) inhibition,^28^ tyrosinase inhibition,^29^ α-amylase inhibition,^30^ and α-glucosidase inhibition,^31^ with slight modifications.^25,32^ For all enzyme inhibition assays, percent inhibition was calculated at various extract concentrations, and IC_50_ values (concentration required to inhibit 50% of enzyme activity) were determined by plotting percent inhibition against extract concentration. All measurements were performed in triplicate.

### Acetylcholinesterase (AChE) inhibition assay

The assay was based on Ellman's method, which measures the hydrolysis of acetylthiocholine iodide by AChE. Extract solutions were mixed with AChE enzyme solution and incubated for 15 minutes. Acetylthiocholine iodide substrate was added, followed by DTNB (5,5′-dithiobis-(2-nitrobenzoic acid)) reagent. The formation of yellow 5-thio-2-nitrobenzoic acid was monitored spectrophotometrically at 412 nm. Galantamine was used as the positive control. Results were expressed as IC_50_ values and galantamine equivalents.

### Butyrylcholinesterase (BChE) inhibition assay

The assay was performed similarly to the AChE assay, using butyrylthiocholine iodide as the substrate and BChE enzyme. Galantamine was used as the positive control. Results were expressed as IC_50_ values and galantamine equivalents.

### Tyrosinase inhibition assay

This assay measures the inhibition of tyrosinase-catalyzed oxidation of l-DOPA (l-3,4-dihydroxyphenylalanine) to dopachrome. Extract solutions were mixed with tyrosinase enzyme solution and incubated for 10 minutes. l-DOPA substrate was added, and the formation of dopachrome was monitored at 492 nm. Kojic acid was used as the positive control. Results were expressed as IC_50_ values and kojic acid equivalents.

### α-Amylase inhibition assay

This assay measures the inhibition of α-amylase-catalyzed hydrolysis of starch. Extract solutions were mixed with α-amylase enzyme solution and incubated for 10 minutes. Starch solution was added as substrate, and the reaction was allowed to proceed for 30 minutes at 37 °C. The reaction was stopped by adding dinitrosalicylic acid (DNS) reagent, and the mixture was heated in a boiling water bath for 5 minutes. After cooling, absorbance was measured at 540 nm. Acarbose was used as the positive control. Results were expressed as IC_50_ values and acarbose equivalents.

### α-Glucosidase inhibition assay

This assay measures the inhibition of α-glucosidase-catalyzed hydrolysis of *p*-nitrophenyl-α-d-glucopyranoside (pNPG). Extract solutions were mixed with α-glucosidase enzyme solution and incubated for 10 minutes. The pNPG substrate was added, and the reaction was allowed to proceed for 30 minutes at 37 °C. The reaction was stopped by adding sodium carbonate solution, and the release of *p*-nitrophenol was measured at 405 nm. Acarbose was used as the positive control. Results were expressed as IC_50_ values and acarbose equivalents.

### Statistical analysis

All experiments were performed in triplicate, and results were expressed as mean ± standard deviation (SD). Statistical analysis was performed using one-way analysis of variance (ANOVA) followed by Tukey's Honest Significant Difference (HSD) post-hoc test to determine significant differences among the three extraction methods (UAE, MAC, and SOE). The level of statistical significance was set at *p* < 0.05. Different superscript letters in tables indicate statistically significant differences among extraction methods for each parameter. Pearson correlation analysis was performed to evaluate relationships among total phenolic content, total flavonoid content, individual phenolic compounds, antioxidant activities, and enzyme inhibitory activities. Correlation coefficients (*r*) were calculated. Statistical analysis was carried out using IBM SPSS Statistics v22.0. To facilitate visualization of the relationships among phytochemical constituents and biological activities, hierarchical cluster analysis (HCA) was additionally performed based on the Pearson correlation matrix. Variables were clustered using the average linkage (UPGMA) algorithm with correlation distance (1 – *r*). The resulting clustered correlation heatmap was generated to identify groups of variables exhibiting similar correlation patterns.

## Results and discussion

### Extraction yield

Extraction yield varied according to the extraction technique, with Soxhlet extraction (SOE) producing the highest yield (12.48%), followed by maceration (MAC, 8.69%) and ultrasonic-assisted extraction (UAE, 7.67%). The superior extraction efficiency of SOE is primarily attributed to prolonged extraction under continuous solvent reflux, which enhances solvent penetration and facilitates exhaustive recovery of extractable constituents from the plant matrix.^5,7^

In contrast, the lower yields obtained with MAC and UAE are likely associated with their milder extraction conditions. Although UAE enhances mass transfer through acoustic cavitation, the relatively short extraction time (30 min) may have limited the recovery of less accessible constituents. Similarly, extraction by maceration relies mainly on passive diffusion at room temperature, resulting in lower extraction efficiency despite prolonged solvent contact.^5,6^

Comparable trends have been reported for other *Genista* species and medicinal plants, where Soxhlet extraction generally provides higher crude extract yields than non-thermal extraction techniques because of continuous solvent renewal and elevated extraction temperature.^1,2^ Nevertheless, a higher extraction yield does not necessarily indicate greater recovery of biologically active phytochemicals, as thermal extraction may also co-extract non-phenolic constituents or promote degradation of thermolabile compounds.^5,7^ Therefore, extraction yield should be interpreted together with phytochemical composition and biological activity rather than as an independent indicator of extraction performance.

The inclusion of Soxhlet extraction in the present study was not intended to recommend this technique to produce functional food ingredients but rather to provide a conventional benchmark against which the performance of milder extraction methods could be evaluated. Although Soxhlet extraction afforded the highest extraction yield, the lower recovery of several flavonoids together with the comparatively weaker biological activities suggests that prolonged heating may have promoted the degradation, oxidation, or structural transformation of thermolabile phytochemicals. These observations are consistent with previous reports indicating that exhaustive hot extraction can increase crude extract yield while simultaneously reducing the abundance of heat-sensitive bioactive constituents.^5–7^

### Total phenolic and total flavonoid content

The total phenolic content (TPC) and total flavonoid content (TFC) of the *G. albida* extracts are presented in Table 1. TPC ranged from 39.84 ± 0.62 to 43.28 ± 0.25 mg GAE per g extract, with UAE exhibiting the highest value, followed by SOE and MAC. However, no statistically significant differences were observed among the extraction methods (*p* > 0.05), indicating that all three extraction techniques recovered comparable amounts of total phenolic constituents. Interestingly, although Soxhlet extraction produced the highest overall extraction yield, it did not yield the highest TPC or TFC, indicating that extraction yield alone is not a reliable indicator of phytochemical quality.

**Table 1:** Extraction yield, total phenolic content and total flavonoid content of methanolic extracts from *G. albida*^a^

Extract	Total phenolic (mg GAE per g extract)	Total flavonoid (mg RE per g extract)	Relative antioxidant capacity index (RACI)
UAE	43.28 ± 0.25^a^	48.41 ± 2.23^ab^	0.39 ± 0.05^a^
MAC	39.84 ± 0.62^a^	50.59 ± 0.29^a^	−0.30 ± 0.03^c^
SOE	41.56 ± 1.93^a^	43.82 ± 1.30^b^	−0.09 ± 0.01^b^

a Values are expressed as mean ± SD (*n* = 3). Different superscript italic letters within the same column indicate significant differences among extracts according to one-way ANOVA followed by Tukey's HSD test (*p* < 0.05). GAE, gallic acid equivalent; RE, rutin equivalent. RACI, Relative Antioxidant Capacity Index.

In contrast, TFC differed significantly among the extracts (*p* < 0.05). MAC yielded the highest flavonoid content (50.59 ± 0.29 mg RE per g extract), followed by UAE (48.41 ± 2.23 mg RE per g extract) and SOE (43.82 ± 1.30 mg RE per g extract). The higher flavonoid recovery achieved by maceration is likely associated with its mild extraction conditions, which minimize thermal degradation of heat-sensitive flavonoids. Conversely, the lower TFC observed in the SOE extract suggests that prolonged exposure to elevated temperature may have promoted degradation or structural transformation of thermolabile flavonoids during extraction.^5–7^

The absence of significant differences in TPC despite marked variation in TFC indicates that extraction strategy affected the composition of phenolic subclasses rather than the overall phenolic content. Since the Folin–Ciocalteu assay estimates the total reducing capacity of a broad range of phenolic compounds, compositional changes among individual phenolics, particularly flavonoids, may not be reflected in total phenolic values. These findings indicate that extraction conditions influence the qualitative composition of phenolic compounds more markedly than the overall phenolic content. Therefore, complementary chromatographic analyses such as LC-MS/MS are essential for achieving a more comprehensive characterization of the phytochemical profile.

Comparable observations have been reported for other *Genista* species, where extraction conditions substantially influenced flavonoid recovery while exerting a comparatively smaller effect on total phenolic content.^1–3^ Nevertheless, quantitative comparisons among studies should be interpreted with caution because phenolic composition is affected by species, geographical origin, plant organ, environmental conditions, solvent system, and extraction protocol. The predominance of flavonoids in *Genista* species, together with their sensitivity to extraction conditions, further highlights the importance of selecting extraction strategies according to the intended biological application.

Overall, these findings indicate that total phenolic content alone is insufficient to explain the phytochemical differences among the extracts. Instead, variations in individual phenolic constituents, particularly flavonoids, are likely responsible for the differences observed among extraction methods and may contribute to the distinct antioxidant and enzyme inhibitory activities of the extracts. Therefore, detailed LC-MS/MS profiling was performed to characterize the individual phenolic compounds underlying these biological properties.

### Phytochemical profiling by LC-MS/MS

**Fig. 1:**
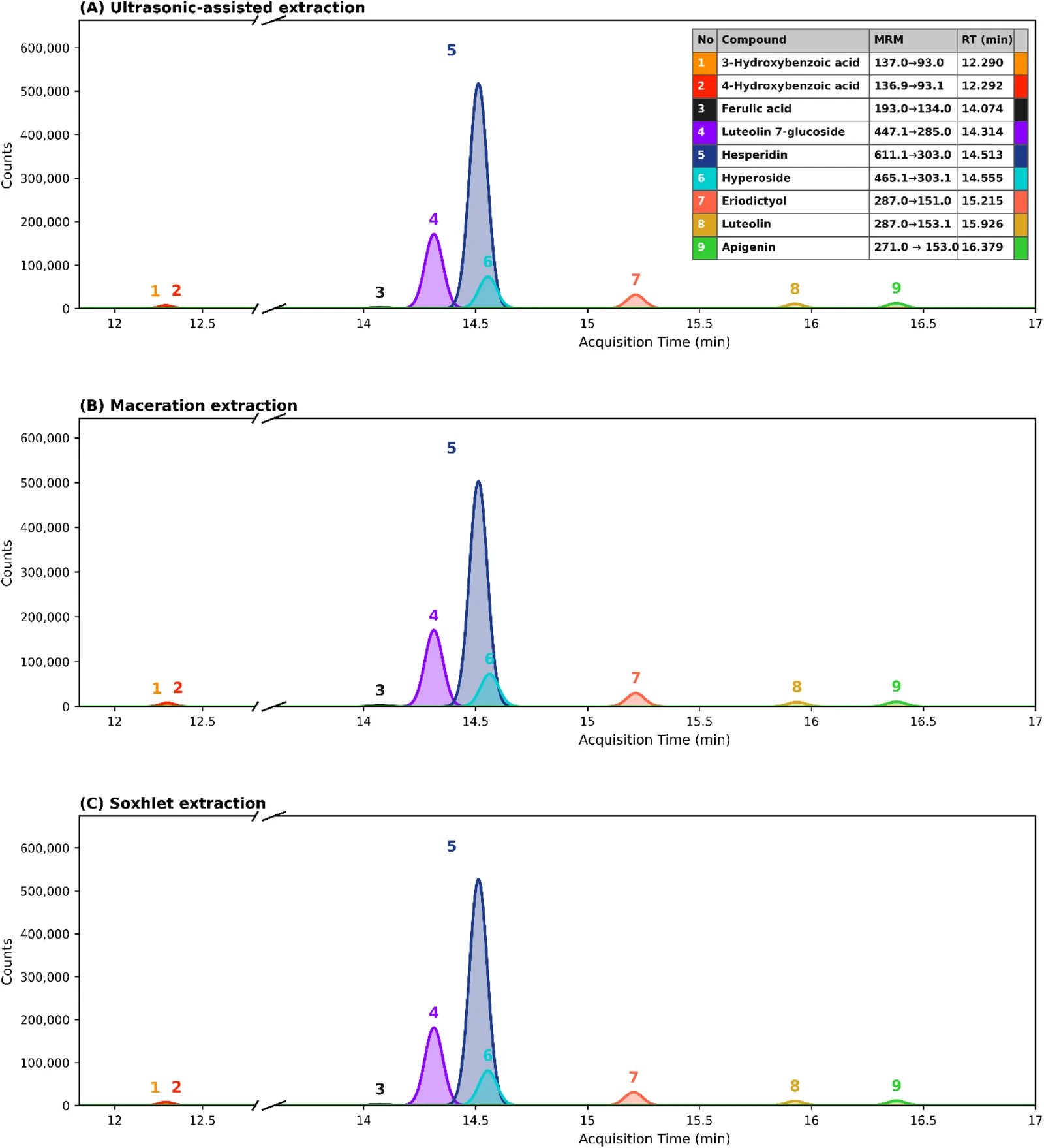
LC-MS/MS MRM chromatograms showing phenolic profiles of *G. albida* extracts prepared by three different extraction methods: (A) ultrasonic-assisted extraction, (B) maceration, and (C) Soxhlet extraction. Peak numbers correspond to the compounds listed in the inset tables within each chromatogram, together with their retention times and MRM transitions. Peak intensities represent MRM signal counts.

LC-MS/MS analysis targeting 31 phenolic compounds resulted in the detection and quantification of 21 compounds in the *G. albida* extracts (Table 2). Compound identification was achieved by comparing retention times and optimized multiple reaction monitoring (MRM) transitions with those of authentic standards using the validated LC–ESI–MS/MS method. Representative chromatograms are presented in Fig. 1, whereas detailed MRM parameters, retention times, calibration data, and analytical characteristics are provided in Supplementary Tables S1 and S2. Ten targeted analytes, namely gallic acid, 3,4-dihydroxyphenylacetic acid, (+)-catechin, pyrocatechol, 2,5-dihydroxybenzoic acid, vanillic acid, 2-hydroxycinnamic acid, pinoresinol, kaempferol, and (–)-epicatechin, were not detected in any extract and were therefore omitted from Table 2.

**Table 2:** Classification and quantification of phenolic compounds identified in methanolic extracts of *G. albida* by LC-MS/MS (µg per g extract).^a^

Chemical class	Compounds	UAE	MAC	SOE
Flavanones	Hesperidin	4567 ± 64^a^	4378 ± 20^b^	4584 ± 24^a^
	Eriodictyol	92.1 ± 0.9^a^	84.9 ± 0.1^b^	87.3 ± 1.7^b^
Flavones	Luteolin	72.9 ± 1.4^a^	68.2 ± 0.3^b^	65.3 ± 0.7^b^
	Apigenin	84.4 ± 0.7^a^	71.5 ± 1.2^b^	72.8 ± 0.2^b^
	Luteolin 7-*O*-glucoside	135 ± 3^ab^	131 ± 1^b^	140 ± 1^a^
	Apigenin 7-*O*-glucoside	43.4 ± 0.4^b^	41.7 ± 1.7^b^	48.0 ± 0.1^a^
Flavonols	Hyperoside	316 ± 1^ab^	302 ± 7^b^	338 ± 7^a^
	Quercetin	8.42 ± 0.25^c^	11.3 ± 0.1^b^	20.0 ± 0.1^a^
Flavanonol	Taxifolin	7.10 ± 0.04^a^	5.78 ± 0.24^b^	6.32 ± 0.01^b^
Hydroxycinnamic acids	Ferulic acid	112 ± 1^a^	101 ± 1^b^	104 ± 3^b^
	*p*-Coumaric acid	31.7 ± 0.7^a^	25.7 ± 0.1^b^	30.0 ± 0.1^a^
	Caffeic acid	12.5 ± 0.1^a^	11.2 ± 0.1^b^	12.2 ± 0.1^a^
	Chlorogenic acid	7.33 ± 0.36^b^	5.69 ± 0.35^c^	9.29 ± 0.11^a^
	Rosmarinic acid	3.34 ± 0.06^b^	3.54 ± 0.35^b^	6.13 ± 0.05^a^
	Sinapic acid	6.10 ± 0.01^a^	5.96 ± 0.33^a^	5.39 ± 0.19^a^
Hydroxybenzoic acids	Protocatechuic acid	35.9 ± 0.4^b^	39.3 ± 1.3^a^	39.6 ± 0.3^a^
	3-Hydroxybenzoic acid	56.5 ± 0.6^b^	62.9 ± 0.4^a^	62.1 ± 0.1^a^
	4-Hydroxybenzoic acid	55.9 ± 0.6^c^	62.5 ± 1.0^a^	59.6 ± 0.1^b^
	Syringic acid	8.29 ± 0.43^a^	8.27 ± 0.28^a^	6.52 ± 0.41^b^
Phenolic aldehyde	Vanillin	18.8 ± 0.2^a^	15.6 ± 0.1^b^	16.3 ± 0.3^b^
Phenylpropanoid glycoside	Verbascoside	2.57 ± 0.23^b^	2.98 ± 0.06^b^	4.70 ± 0.34^a^

a Values are expressed as mean ± SD (*n* = 3). Different superscript italic letters within the same row indicate significant differences among extracts according to one-way ANOVA followed by Tukey's HSD test (*p* < 0.05).

The phytochemical profile of *G. albida* was dominated by flavonoids, particularly flavanone and flavonol glycosides, together with appreciable amounts of hydroxycinnamic and hydroxybenzoic acids. Among the identified constituents, hesperidin was by far the predominant compound in all extracts (4378–4584 µg per g extract), followed by hyperoside, luteolin 7-*O*-glucoside, ferulic acid, eriodictyol, apigenin, and luteolin. The predominance of hesperidin is consistent with recent phytochemical investigations on other *Genista* species, suggesting that this flavanone glycoside represents one of the characteristic metabolites of the genus.^2,4^ Given its well-documented antioxidant and α-glucosidase inhibitory activities, hesperidin is likely to contribute substantially to the biological activities observed for *G. albida* extracts.^33^

Although hesperidin remained the dominant constituent regardless of extraction method, individual phenolic compounds exhibited distinct extraction patterns. Extraction selectivity differed markedly among the extraction methods. UAE favored the recovery of several flavonoid aglycones and hydroxycinnamic acids, including eriodictyol, apigenin, luteolin, ferulic acid, *p*-coumaric acid, caffeic acid, vanillin, and taxifolin. In contrast, SOE preferentially extracted flavonoid glycosides and other polar phenolics, such as hyperoside, luteolin 7-*O*-glucoside, apigenin 7-*O*-glucoside, chlorogenic acid, rosmarinic acid, verbascoside, and quercetin. MAC generally produced intermediate concentrations for most compounds but showed comparatively higher levels of several hydroxybenzoic acids, including protocatechuic acid, 3-hydroxybenzoic acid, and 4-hydroxybenzoic acid. These findings demonstrate that extraction strategy influenced the qualitative composition of phenolic subclasses more strongly than the overall phenolic content, supporting the observations obtained from the TPC and TFC assays.

The observed extraction selectivity can largely be explained by differences in extraction conditions. Ultrasonic-assisted extraction favors rapid disruption of plant tissues and enhanced mass transfer under relatively mild temperatures, facilitating the recovery of several flavonoid aglycones and hydroxycinnamic acids while minimizing thermal degradation. In contrast, the continuous solvent reflux employed during Soxhlet extraction appears to improve the extraction of several glycosylated flavonoids and more tightly bound phenolic constituents. Nevertheless, the lower total flavonoid content observed for SOE suggests that prolonged heating may simultaneously promote degradation or transformation of certain thermolabile flavonoids, indicating that higher extraction efficiency does not necessarily correspond to greater preservation of all bioactive metabolites.^5–7^

The overall phytochemical composition agrees well with previous reports describing *Genista* species as rich sources of flavonoids and phenolic acids, although quantitative differences among species are expected because phytochemical accumulation is influenced by genetic background, environmental conditions, geographical origin, developmental stage, extraction solvent, and extraction protocol.^1–3^ The predominance of hesperidin together with hyperoside, luteolin derivatives, apigenin derivatives, eriodictyol, and ferulic acid further expands the phytochemical knowledge of *G. albida*, an endemic Turkish species for which comprehensive metabolite profiling has previously been unavailable.

Importantly, the identified phytochemical profile provides a plausible explanation for the biological activities observed in the present study. Hesperidin, hyperoside, luteolin derivatives, eriodictyol, apigenin, ferulic acid, and caffeic acid have all been reported to possess strong antioxidant properties and inhibitory effects against carbohydrate-hydrolyzing enzymes, particularly α-glucosidase, while several flavonoids also exhibit cholinesterase inhibitory activity.^1,2,33^ The combined presence of these metabolites, rather than the abundance of a single compound, is therefore likely responsible for the multifunctional biological properties of *G. albida*. These observations support the subsequent antioxidant and enzyme inhibition results and further emphasize the importance of extraction strategy in maximizing the recovery of bioactive phytochemicals for functional food and nutraceutical applications.

The observed biological activities can be better understood by considering the structural characteristics and well-established biofunctional properties of the identified phenolic subclasses. Hydroxycinnamic acids, including ferulic, caffeic, chlorogenic, *p*-coumaric, rosmarinic, and sinapic acids, are recognized for their strong electron-donating capacity, hydrogen atom transfer ability, and free radical scavenging activity, which are largely attributed to hydroxyl substitution on the aromatic ring and resonance stabilization of phenoxyl radicals.^34^ Flavones, represented by luteolin and apigenin together with their glycosides, have been reported to inhibit carbohydrate-hydrolyzing enzymes through hydrogen bonding and π–π interactions with catalytic residues at the enzyme active site, thereby contributing to α-glucosidase and α-amylase inhibition.^35,36^ Flavonols, including hyperoside and quercetin, exhibit potent antioxidant activity owing to their catechol B-ring structure and conjugated double-bond system, which facilitate electron transfer, radical stabilization, and metal ion chelation.^34^ In addition, flavanones such as hesperidin and eriodictyol have been associated with antioxidant, anti-inflammatory, and glucose-modulating effects, supporting the multifunctional biological profile of the *G. albida* extracts.^33^ Therefore, the observed biological activities are most likely the result of complementary and potentially synergistic interactions among multiple phenolic subclasses rather than the action of a single dominant constituent.

A limitation of the present phytochemical analysis is that the LC-MS/MS method was based on a previously validated targeted analytical panel comprising 31 selected phenolic compounds,^16^ rather than a metabolite library specifically developed for the *Genista* genus. Consequently, several isoflavonoids that have been reported in certain *Genista* species, including genistein and its derivatives,^2^ were not included in the targeted analyte list and therefore could not be evaluated in the present study. Future investigations employing *Genista*-specific targeted methods or untargeted high-resolution metabolomic approaches would provide a more comprehensive characterization of the phytochemical profile of *G. albida*.

### Antioxidant activity

The antioxidant capacity of *G. albida* extracts was comprehensively evaluated using six complementary *in vitro* assays representing different antioxidant mechanisms, including total antioxidant capacity, reducing power, radical scavenging activity, and metal chelation (Tables 3 and 4). To facilitate an integrated comparison among extraction methods, the Relative Antioxidant Capacity Index (RACI) was calculated from the standardized results of all assays. Among the extracts, UAE exhibited the highest overall antioxidant performance (RACI = 0.39), followed by SOE (−0.09) and MAC (−0.30), indicating that ultrasonic-assisted extraction provided the most favorable balance of antioxidant activities. This superior performance is consistent with the higher recovery of several antioxidant phenolics, particularly ferulic acid, eriodictyol, caffeic acid, and luteolin, as revealed by LC-MS/MS profiling.

**Table 3:** EC_50_ and IC_50_ values (mg mL^−1^) of the antioxidant and enzyme inhibitory activities of methanolic extracts from *G. albida*.^a^

Assay	UAE	MAC	SOE	Standard
Phosphomolybdenum (EC_50_)	0.78 ± 0.01^b^	0.85 ± 0.02^c^	0.84 ± 0.01^c^	0.39 ± 0.02^a^
CUPRAC (EC_50_)	1.45 ± 0.08^b^	1.60 ± 0.12^b^	1.52 ± 0.01^b^	0.17 ± 0.01^a^
FRAP (EC_50_)	0.53 ± 0.01^b^	0.54 ± 0.01^b^	0.55 ± 0.02^b^	0.049 ± 0.001^a^
DPPH (IC_50_)	4.81 ± 0.04^c^	4.82 ± 0.25^c^	4.28 ± 0.04^b^	0.22 ± 0.02^a^
ABTS (IC_50_)	1.46 ± 0.09^b^	1.48 ± 0.05^b^	1.31 ± 0.02^b^	0.16 ± 0.01^a^
Metal chelating (IC_50_)	1.05 ± 0.02^b^	1.03 ± 0.00^b^	1.06 ± 0.01^b^	0.014 ± 0.001^a^
AChE (IC_50_)	1.23 ± 0.03^c^	1.13 ± 0.02^b^	1.19 ± 0.01^bc^	0.0034 ± 0.0003^a^
BChE (IC_50_)	1.04 ± 0.02^c^	1.06 ± 0.01^c^	0.96 ± 0.02^b^	0.0054 ± 0.0004^a^
Tyrosinase (IC_50_)	1.46 ± 0.01^b^	1.46 ± 0.005^b^	1.49 ± 0.01^b^	0.083 ± 0.003^a^
α-Amylase (IC_50_)	4.36 ± 0.05^b^	5.36 ± 0.02^d^	4.51 ± 0.03^c^	1.50 ± 0.01^a^
α-Glucosidase (IC_50_)	0.66 ± 0.01^b^	0.64 ± 0.003^b^	0.63 ± 0.004^b^	0.96 ± 0.02^a^

a Values are expressed as mean ± SD (*n* = 3). Different superscript letters within the same row indicate significant differences among extracts according to one-way ANOVA followed by Tukey's HSD test (*p* < 0.05). IC_50_ represents the concentration required to achieve 50% inhibition. EC_50_ represents the concentration producing an absorbance of 0.500 in the phosphomolybdenum, CUPRAC, and FRAP assays. Standard compounds: Trolox (phosphomolybdenum, DPPH, ABTS, CUPRAC and FRAP), EDTA (metal chelating), galantamine (AChE and BChE), kojic acid (tyrosinase), and acarbose (α-amylase and α-glucosidase). na, no activity.

**Table 4:** Antioxidant and enzyme inhibitory activities of Methanolic Extracts from *G. albida* expressed as standard equivalent activities^a^

Assay	Unit	UAE	MAC	SOE
Phosphomolybdenum	mg TE per g extract	500.64 ± 4.50^a^	460.12 ± 9.60^b^	466.91 ± 6.00^b^
DPPH	mg TE per g extract	47.84 ± 0.42^a^	47.74 ± 2.52^a^	53.68 ± 0.56^a^
ABTS	mg TE per g extract	110.59 ± 6.80^a^	108.54 ± 3.68^a^	123.21 ± 1.45^a^
CUPRAC	mg TE per g extract	116.05 ± 6.11^a^	104.73 ± 8.01^a^	110.49 ± 0.95^a^
FRAP	mg TE per g extract	93.21 ± 1.47^a^	90.41 ± 0.86^a^	89.95 ± 3.15^a^
Metal chelating	mg EDTAE per g extract	13.93 ± 0.27^a^	14.17 ± 0.05^a^	13.70 ± 0.13^a^
AChE	mg GALAE per g extract	2.68 ± 0.07^b^	2.92 ± 0.05^a^	2.77 ± 0.03^ab^
BChE	mg GALAE per g extract	5.18 ± 0.09^b^	5.10 ± 0.04^b^	5.61 ± 0.10^a^
Tyrosinase	mg KAE per g extract	55.33 ± 0.38^a^	55.35 ± 0.19^a^	54.46 ± 0.53^a^
α-Amylase	mg ACE per g extract	344.44 ± 3.77^a^	280.15 ± 0.80^c^	333.18 ± 2.54^b^
α-Glucosidase	mg ACE per g extract	846.26 ± 12.23^b^	872.90 ± 4.13^ab^	884.99 ± 5.42^a^

a Values are expressed as mean ± SD (*n* = 3). Different superscript letters within the same row indicate significant differences among extracts according to one-way ANOVA followed by Tukey's HSD test (*p* < 0.05). TE, Trolox equivalent; EDTAE, ethylenediaminetetraacetic acid equivalent; GALAE, galantamine equivalent; KAE, kojic acid equivalent; ACE, acarbose equivalent; na, no activity.

Reducing capacity assays showed a consistent advantage for UAE. In the phosphomolybdenum assay, UAE exhibited significantly greater total antioxidant capacity than both MAC and SOE (*p* < 0.05), while slightly stronger reducing power was also observed in the CUPRAC and FRAP assays, although these differences were not statistically significant. Correlation analysis indicated that ferulic acid and eriodictyol were the individual compounds most strongly associated with reducing capacity, suggesting that these phenolics are major contributors to the electron-donating ability of the extracts. The higher abundance of these compounds in UAE provides a plausible explanation for its superior reducing performance. These findings are also consistent with the relatively higher total phenolic content determined for this extract, supporting the important contribution of phenolic constituents to electron-transfer-based antioxidant mechanisms.

In contrast, SOE demonstrated the strongest radical scavenging activity in both DPPH and ABTS assays, although statistically significant differences among extraction methods were observed only for DPPH. The enhanced radical scavenging capacity of SOE is likely associated with its higher concentrations of hyperoside and luteolin 7-*O*-glucoside, both of which showed strong positive correlations with radical scavenging activity. These flavonoids possess structural characteristics favorable for hydrogen atom donation and electron transfer, supporting their contribution to free radical neutralization. Although hesperidin was the predominant constituent of all extracts, its contribution to radical scavenging appears to be less pronounced than that of flavonol and flavone derivatives, indicating that antioxidant activity depends not only on compound abundance but also on molecular structure.

Metal chelating activity differed only marginally among the extraction methods, with no statistically significant differences observed. Correlation analysis suggested a moderate relationship between flavonoid content and metal chelation, whereas hesperidin showed a strong negative correlation with this assay. This observation is consistent with the structural characteristics of hesperidin, which lacks the catechol and 3-hydroxyl functionalities generally associated with efficient metal binding. Moreover, the strong negative correlations between metal chelating activity and radical scavenging assays indicate that these antioxidant mechanisms are governed by different groups of phenolic constituents rather than by a common set of metabolites.

Overall, the antioxidant profile of *G. albida* reflects the complementary contribution of multiple phenolic constituents rather than the dominance of a single compound. LC-MS/MS analysis demonstrated that UAE preferentially recovered ferulic acid, eriodictyol, caffeic acid, and luteolin, whereas SOE favored hyperoside and luteolin glycosides, explaining the distinct antioxidant patterns observed among extraction methods. Similar relationships between phenolic composition and antioxidant performance have been reported for other *Genista* species, confirming that flavonoids and phenolic acids are the principal contributors to the antioxidant potential of this genus.^1–3,37^ The complementary behavior observed among different antioxidant assays further emphasizes the importance of employing multiple analytical approaches when evaluating plant-derived antioxidants.

Collectively, these findings demonstrate that extraction strategy substantially influences antioxidant performance by selectively enriching different phenolic subclasses. While UAE provided the highest overall antioxidant capacity according to RACI, SOE was particularly effective in enhancing radical scavenging activity through improved recovery of flavonoid glycosides. These findings provide a mechanistic explanation for the enzyme inhibitory activities discussed below, where several of the same phenolic constituents are implicated in α-amylase and particularly α-glucosidase inhibition.

### Enzyme inhibition activities

The enzyme inhibitory activities of *G. albida* extracts were evaluated against five therapeutically relevant enzymes, namely acetylcholinesterase (AChE), butyrylcholinesterase (BChE), tyrosinase, α-amylase, and α-glucosidase (Tables 3 and 4). Overall, extraction strategy markedly influenced the enzyme inhibition profile by selectively enriching different classes of phenolic constituents. Notably, all three extracts exhibited significantly stronger α-glucosidase inhibitory activity than the reference inhibitor acarbose, representing the most noteworthy biological finding of the present study and highlighting the potential of *G. albida* as a promising source of natural antidiabetic phytochemicals.

The cholinesterase inhibitory activities differed significantly according to extraction method. MAC exhibited the strongest AChE inhibition, whereas SOE showed the highest BChE inhibitory activity. Although all extracts were less potent than galantamine, a clear preference toward BChE inhibition was observed across all extraction methods, with BChE IC_50_ values consistently lower than those for AChE. This finding is noteworthy because BChE activity becomes increasingly important during the progression of Alzheimer's disease as AChE activity declines.^38,39^ Correlation analysis further demonstrated that AChE inhibition was primarily associated with hesperidin, eriodictyol, ferulic acid, and apigenin, whereas BChE inhibition was closely related to hyperoside and luteolin 7-*O*-glucoside. These relationships agree well with the LC-MS/MS results, indicating that extraction-dependent changes in flavonoid composition directly influenced cholinesterase inhibitory activity. Similar cholinesterase inhibitory properties have previously been reported for other *Genista* species, supporting the genus as a valuable source of neuroprotective phytochemicals.^2,4^

Tyrosinase inhibition showed minimal variation among the three extraction methods, suggesting that the compounds responsible for this activity were extracted with comparable efficiency regardless of extraction technique. The similar IC_50_ values obtained for UAE, MAC, and SOE indicate that tyrosinase inhibition is relatively independent of extraction selectivity under the present experimental conditions. Correlation analysis suggested that this activity was associated with flavonoids possessing metal-binding structural features, consistent with the well-established mechanism whereby polyphenols inhibit tyrosinase through copper chelation at the enzyme active site.^29,34^ Although the inhibitory activities were lower than that of kojic acid, the results confirm that *G. albida* contains naturally occurring compounds capable of modulating tyrosinase activity.

The inhibitory activities against carbohydrate-hydrolyzing enzymes further demonstrated the influence of extraction strategy on biological performance. UAE produced the strongest α-amylase inhibition, which was positively associated with total phenolic content together with hesperidin, ferulic acid, and eriodictyol. These compounds have previously been recognized as important contributors to glucose homeostasis through multiple mechanisms, including inhibition of digestive enzymes and improvement of glucose metabolism.^33,40^ In contrast, α-glucosidase inhibition was remarkably consistent among all extracts despite differences in phytochemical composition. More importantly, every extract exhibited significantly stronger inhibitory activity than acarbose, with IC_50_ values ranging from 0.63 to 0.66 mg mL^−1^ compared with 0.96 mg mL^−1^ for the reference inhibitor. This outstanding activity represents the most significant biological finding of the present study and strongly supports the potential use of *G. albida* as a natural source of antidiabetic ingredients.

Correlation analysis indicated that α-glucosidase inhibition was strongly associated with luteolin, apigenin, and ferulic acid, suggesting that flavone aglycones are the principal contributors to this activity. Previous studies have demonstrated that luteolin and apigenin effectively inhibit α-glucosidase by interacting with catalytic residues within the enzyme active site through competitive or mixed inhibition mechanisms.^35,36^ Nevertheless, the comparable inhibitory activities observed among the three extraction methods despite quantitative differences in individual compounds indicate that α-glucosidase inhibition most likely results from synergistic interactions among multiple phenolic constituents rather than from the action of a single metabolite. Such synergistic behavior is frequently reported for phenolic-rich plant extracts and represents an important advantage for functional food applications.

Overall, the enzyme inhibition profile closely reflected the phytochemical composition determined by LC-MS/MS analysis. UAE preferentially recovered phenolic compounds associated with antioxidant activity and α-amylase inhibition, MAC produced extracts with superior AChE inhibitory activity, whereas SOE favored compounds contributing to BChE inhibition while maintaining potent α-glucosidase inhibition. Collectively, these findings demonstrate that extraction strategy influences the biological functionality of *G. albida* through selective enrichment of different phenolic subclasses. The combination of pronounced antioxidant capacity, potent enzyme inhibitory activities, and particularly the superior α-glucosidase inhibition relative to acarbose positions this endemic Turkish species as a promising candidate for the development of functional foods and nutraceuticals aimed at the dietary management of postprandial hyperglycemia.

The enzyme models used in the present study are widely accepted and extensively employed in preliminary screening studies of natural products because they provide reproducible, commercially available, and well-characterized systems for evaluating enzyme inhibitory potential.^15,28^ Although these enzymes are not of human origin, they have been extensively used as standardized experimental models for the comparative assessment of plant-derived enzyme inhibitors. Nevertheless, further validation using human enzymes and *in vivo* models will be necessary before extrapolating the present findings to clinical or functional food applications.

### Pearson correlation analysis and hierarchical clustering

**Fig. 2:**
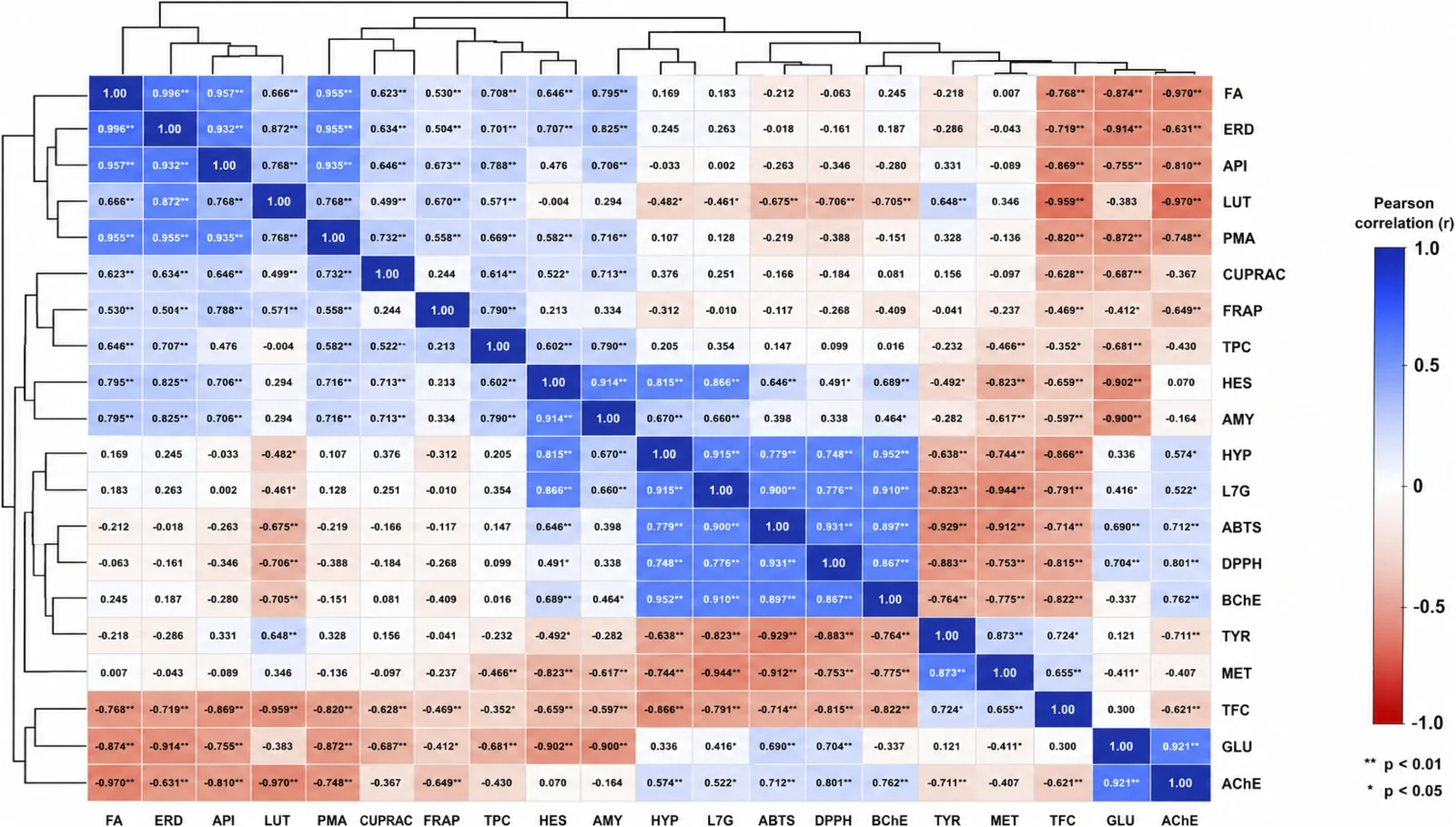
Hierarchically clustered Pearson correlation heatmap illustrating the relationships among antioxidant activities, enzyme inhibitory activities, total phenolic and flavonoid contents, and major phenolic compounds identified in Genista albida extracts. Hierarchical clustering analysis (HCA) was performed using the average linkage (UPGMA) method based on the Pearson correlation distance (1 – *r*). Blue and red colors represent positive and negative Pearson correlation coefficients, respectively, while color intensity reflects the strength of the correlation. Correlation coefficients (*r*) are shown within each cell. Significant correlations are indicated as *p* < 0.05 (*) and *p* < 0.01 (**). PMA, phosphomolybdenum assay; CUPRAC, cupric ion reducing antioxidant capacity; FRAP, ferric reducing antioxidant power; TPC, total phenolic content; HES, hesperidin; AMY, α-amylase inhibition; HYP, hyperoside; L7G, luteolin 7-*O*-glucoside; ABTS, 2,2′-azinobis-(3-ethylbenzothiazoline-6-sulfonic acid) radical scavenging assay; DPPH, 2,2-diphenyl-1-picrylhydrazyl radical scavenging assay; BChE, butyrylcholinesterase inhibition; TYR, tyrosinase inhibition; MET, metal chelating activity; TFC, total flavonoid content; GLU, α-glucosidase inhibition; AChE, acetylcholinesterase inhibition; FA, ferulic acid; ERD, eriodictyol; API, apigenin; LUT, luteolin.

Pearson correlation analysis was performed using all phenolic compounds identified by LC-MS/MS. For clarity of visualization, Fig. 2 presents the major correlation patterns, whereas the complete Pearson correlation matrix including all identified compounds is provided in Supplementary Table S3. To facilitate the interpretation of these complex relationships, hierarchical cluster analysis (HCA) was applied to the Pearson correlation matrix, and the results were visualized as a clustered heatmap (Fig. 2). The HCA clearly grouped variables according to similarities in their correlation profiles, allowing the identification of distinct phytochemical–bioactivity clusters.

The clustered heatmap highlighted several biologically meaningful relationships. Ferulic acid, eriodictyol, and apigenin clustered together with phosphomolybdenum activity, CUPRAC, FRAP, and α-amylase inhibition, suggesting that these compounds collectively contribute to electron transfer-based antioxidant mechanisms and carbohydrate-hydrolyzing enzyme inhibition. Conversely, hyperoside and luteolin 7-*O*-glucoside formed a separate cluster closely associated with DPPH, ABTS, and butyrylcholinesterase inhibition, indicating a stronger contribution to radical scavenging activity and cholinesterase inhibition.

Interestingly, α-glucosidase inhibition showed a distinct clustering pattern, being more closely associated with luteolin and apigenin than with total phenolic or flavonoid contents. This observation supports the hypothesis that α-glucosidase inhibition depends primarily on the qualitative composition of phenolic compounds rather than their overall abundance. Overall, the HCA confirmed that extraction-dependent changes in phytochemical composition are closely reflected in the multifunctional biological activities of *G. albida* extracts.

A limitation of the present study is that methanol was selected as the extraction solvent to maximize the analytical recovery of phenolic compounds and ensure compatibility with LC-MS/MS analysis.^5,6,8^ Although this approach is widely accepted for phytochemical profiling, methanol is not suitable for the preparation of food-grade extracts. Therefore, future studies should evaluate food-grade solvents, particularly ethanol and hydroethanolic mixtures, to validate the present findings under conditions more directly applicable to functional food and nutraceutical development.^6,8^

## Conclusion

The present study demonstrates that the extraction strategy plays a significant role in determining both the phytochemical composition and the multifunctional biological activities of *G. albida* extracts. While Soxhlet extraction provided the highest extraction yield, ultrasonic-assisted extraction was superior in terms of total phenolic content and overall antioxidant capacity, whereas maceration yielded the highest flavonoid content and exhibited the strongest acetylcholinesterase inhibitory activity. These findings indicate that no single extraction method is universally optimal, and the choice of extraction technique should be guided by the intended biological application.

Comprehensive LC–ESI–MS/MS profiling revealed hesperidin as the predominant phenolic constituent in all extracts, together with considerable amounts of hyperoside, luteolin 7-*O*-glucoside, ferulic acid, eriodictyol, apigenin, and luteolin. Differences in phytochemical composition were consistently reflected in distinct antioxidant and enzyme inhibitory activity profiles, demonstrating that extraction selectively enriches specific groups of bioactive compounds. Furthermore, hierarchical clustering analysis provided an integrated visualization of the phytochemical–bioactivity relationships and confirmed that extraction-induced variations in phenolic composition were closely associated with distinct antioxidant and enzyme inhibitory activity profiles.

Among all biological activities evaluated, the most remarkable finding was the potent α-glucosidase inhibitory activity exhibited by all three extracts, which surpassed that of the reference inhibitor acarbose. Considering their complementary antioxidant and enzyme inhibitory properties, *G. albida* extracts represent a promising natural source of multifunctional phytochemicals with potential applications in functional foods, nutraceuticals, and the dietary management of postprandial hyperglycemia and oxidative stress-related disorders. Future studies focusing on bioavailability, mechanism of action, and *in vivo* efficacy will further clarify their therapeutic and functional potential.

## Author contributions

Aysegul Dinc: investigation, data curation, formal analysis, writing – original draft. Ilkay Kir: methodology, investigation, data curation, formal analysis, writing – original draft Cengiz Sarikurkcu: writing – original draft, validation, methodology, investigation, data curation, conceptualization, writing – review and editing.

## Conflicts of interest

All authors declare that they have no conflicts of interest.

## Supplementary Material

RA-016-D6RA06022F-s001

## Data Availability

The data supporting the findings of this study are available within the article and its supplementary information (SI). Additional data are available from the corresponding author upon reasonable request. Supplementary information is available. See DOI: https://doi.org/10.1039/d6ra06022f.
