# Supplementary material for: Extraction strategy influences the phytochemical composition and multifunctional biological activities of Genista albida: implications for functional food development
Source: RSC Adv. 2026 Aug 10;16(40):46366–78. doi: 10.1039/d6ra06022f (PMC13455645; doi:10.1039/d6ra06022f)
Supplement: Supplementary file 1 [file RA-016-D6RA06022F-s001.pdf]

Supplementary Information (SI) for RSC Advances.
This journal is © The Royal Society of Chemistry 2026

Supplementary Materials

Supplementary Table S1. ESI–MS/MS Parameters and analytical characteristics for the Analysis of Target Analytes by MRM Negative and Positive Ionization Mode

Target compounds	Rt (min)	Precursor ion	MRM1 (CE, V)	MRM2 (CE, V)
Compounds analyzed by NI mode				
Gallic acid	8.891	168.9 $[M-H]^-$	125.0 (10)	–
Protocatechuic acid	10.818	152.9 $[M-H]^-$	108.9 (12)	–
3,4-Dihydroxyphenylacetic acid	11.224	167.0 $[M-H]^-$	123.0 (2)	–
(+)-Catechin	11.369	289.0 $[M-H]^-$	245.0 (6)	202.9 (12)
Pyrocatechol	11.506	109.0 $[M-H]^-$	90.6 (18)	52.9 (16)
2,5-Dihydroxybenzoic acid	12.412	152.9 $[M-H]^-$	109.0 (10)	–
4-Hydroxybenzoic acid	12.439	136.9 $[M-H]^-$	93.1 (14)	–
Caffeic acid	12.841	179.0 $[M-H]^-$	135.0 (12)	–
Vanillic acid	12.843	166.9 $[M-H]^-$	151.8 (10)	122.6 (6)
Syringic acid	12.963	196.9 $[M-H]^-$	181.9 (8)	152.8 (6)
3-Hydroxybenzoic acid	13.259	137.0 $[M-H]^-$	93.0 (6)	–
Vanillin	13.397	151.0 $[M-H]^-$	136.0 (10)	–
Verbascoside	13.589	623.0 $[M-H]^-$	461.0 (26)	160.8 (36)
Taxifolin	13.909	303.0 $[M-H]^-$	285.1 (2)	125.0 (14)
Sinapic acid	13.992	222.9 $[M-H]^-$	207.9 (6)	163.8 (6)
p-Coumaric acid	14.022	162.9 $[M-H]^-$	119.0 (12)	–
Ferulic acid	14.120	193.0 $[M-H]^-$	177.8 (8)	134.0 (12)
Luteolin 7-glucoside	14.266	447.1 $[M-H]^-$	285.0 (24)	–

Rosmarinic acid	14.600	359.0 [M − H]−	196.9 (10)	160.9 (10)
2-Hydroxycinnamic acid	15.031	162.9 [M − H]−	119.1 (10)	–
Pinoresinol	15.118	357.0 [M − H]−	151.0 (12)	135.7 (34)
Eriodictyol	15.247	287.0 [M − H]−	151.0 (4)	134.9 (22)
Quercetin	15.668	301.0 [M − H]−	178.6 (10)	151.0 (16)
Kaempferol	16.236	285.0 [M − H]−	242.8 (16)	229.1 (18)
Compounds analyzed by PI mode				
Chlorogenic acid	11.802	355.0 [M + H]+	163.0 (10)	–
(−)-Epicatechin	12.458	291.0 [M + H]+	139.1 (12)	122.9 (36)
Hesperidin	14.412	611.1 [M + H]+	449.2 (4)	303.0 (20)
Hyperoside	14.506	465.1 [M + H]+	303.1 (8)	–
Apigenin 7-glucoside	14.781	433.1 [M + H]+	271.0 (18)	–
Luteolin	15.923	287.0 [M + H]+	153.1 (34)	135.1 (36)
Apigenin	16.382	271.0 [M + H]+	153.0 (34)	119.1 (36)

R_t, retention time; NI, negative ion; and PI, positive ion.

Supplementary Table S2. Calibration curves and sensitivity properties of the method

Compounds	Linearity and sensitivity characteristics				
	Range (μg/L)	Linear equation	R^2	LOD (μg/L)	LOQ (μg/L)
Gallic acid	5–500	y = 4.82x − 26.48	0.9988	1.46	4.88
Protocatechuic acid	2.5–500	y = 5.65x − 9.99	0.9990	1.17	3.88
3,4-Dihydroxyphenylacetic acid	5–500	y = 5.13x − 12.39	0.9990	1.35	4.51
(+)-Catechin	10–500	y = 1.45x + 1.95	0.9974	3.96	13.20
Pyrocatechol	25–400	y = 0.11x − 0.52	0.9916	9.62	32.08
Chlorogenic acid	1–500	y = 12.14x + 32.34	0.9995	0.55	1.82
2,5-Dihydroxybenzoic acid	5–500	y = 3.79x − 14.12	0.9980	2.12	7.08
4-Hydroxybenzoic acid	5–500	y = 7.62x + 22.79	0.9996	1.72	5.72
(−)-Epicatechin	5–500	y = 9.11x − 9.99	0.9971	1.85	6.18
Caffeic acid	5–500	y = 11.09x + 16.73	0.9997	3.15	10.50
Vanillic acid	10–500	y = 0.49x − 1.61	0.9968	2.56	8.54
Syringic acid	10–500	y = 0.74x − 1.54	0.9975	3.75	12.50
3-Hydroxybenzoic acid	5–500	y = 3.69x − 12.29	0.9991	1.86	6.20
Vanillin	50–500	y = 2.02x + 135.49	0.9926	15.23	50.77
Verbascoside	2.5–500	y = 8.59x − 28.05	0.9988	0.82	2.75
Taxifolin	5–500	y = 12.32x + 9.98	0.9993	1.82	6.05
Sinapic acid	5–500	y = 2.09x − 6.79	0.9974	2.64	8.78
p-Coumaric acid	5–500	y = 17.51x + 53.73	0.9997	1.93	6.44
Ferulic acid	5–500	y = 3.32x − 4.30	0.9992	1.43	4.76
Luteolin 7-glucoside	1–500	y = 45.25x + 156.48	0.9996	0.45	1.51
Hesperidin	5–500	y = 5.98x + 0.42	0.9993	1.73	5.77
Hyperoside	2.5–500	y = 16.32x − 1.26	0.9998	0.99	3.31
Rosmarinic acid	1–500	y = 9.82x − 17.98	0.9989	0.57	1.89
Apigenin 7-glucoside	1–500	y = 21.33x − 31.69	0.9983	0.41	1.35
2-Hydroxycinnamic acid	1–500	y = 16.72x − 26.94	0.9996	0.61	2.03
Pinoresinol	10–500	y = 0.80x − 2.69	0.9966	3.94	13.12
Eriodictyol	2.5–500	y = 14.24x − 0.50	0.9998	0.80	2.68
Quercetin	5–500	y = 14.68x − 18.25	0.9997	1.23	4.10
Luteolin	5–500	y = 8.96x + 26.80	0.9992	1.34	4.46
Kaempferol	10–500	y = 0.82x − 3.06	0.9959	3.30	10.99
Apigenin	2.5–500	y = 11.29x + 38.05	0.9987	0.96	3.20

LOD and LOQ: limit of detection and limit of quantification, respectively. All calibration curves exhibited excellent linearity ($R^2 > 0.99$), confirming the suitability of the validated LC–MS/MS method for quantitative analysis.

Structural confirmation of major phenolic constituents by LC–ESI–MS/MS

The identities of the major phenolic constituents detected in *G. albida* extracts were confirmed by comparing their retention times and characteristic MRM transitions with

those of authentic reference standards analyzed under identical chromatographic conditions. The observed fragmentation patterns were consistent with previously published LC–ESI–MS/MS studies, providing additional confidence for compound identification.

Hesperidin

Hesperidin was identified in the positive ionization mode by its protonated molecular ion at m/z 611.1 ([M+H]+) together with the characteristic product ions at m/z 449.2 and m/z 303.0. These fragment ions correspond to the sequential loss of the rutinose moiety followed by formation of the hesperetin aglycone, which is characteristic of hesperidin fragmentation reported previously.

Hyperoside

Hyperoside was identified from its protonated precursor ion at m/z 465.1, producing the diagnostic fragment ion at m/z 303.1, corresponding to cleavage of the galactoside moiety and formation of the quercetin aglycone.

Luteolin-7-*O*-glucoside

Luteolin-7-*O*-glucoside produced a deprotonated molecular ion at m/z 447.1 ([M−H]−) and the characteristic fragment ion at m/z 285.0, resulting from the neutral loss of glucose and formation of luteolin.

Apigenin-7-*O*-glucoside

Apigenin-7-*O*-glucoside was identified by the protonated molecular ion at m/z 433.1 together with the fragment ion at m/z 271.0, representing the corresponding apigenin aglycone after cleavage of the glucose residue.

Ferulic acid

Ferulic acid was detected in the negative ionization mode with a precursor ion at m/z 193.0 and characteristic fragment ions at m/z 177.8 and m/z 134.0, which agree well with previously reported fragmentation pathways.

Luteolin

Luteolin exhibited a protonated molecular ion at m/z 287.0 with characteristic product ions at m/z 153.1 and m/z 135.1, corresponding to the typical retro-Diels–Alder fragmentation of flavones.

Overall, the observed retention times, optimized MRM transitions, and characteristic fragmentation patterns were consistent with authentic reference standards and previously reported LC–ESI–MS/MS fragmentation data, confirming the reliable identification of the major phenolic constituents detected in *G. albida* extracts (Cuyckens and Claeys, 2004; Hvattum, 2002).

References

Cuyckens, F and Claeys, M Mass spectrometry in the structural analysis of flavonoids. Journal of Mass Spectrometry. *39*: 1–15 (2004).

Hvattum, E Determination of phenolic compounds in rose hip (*Rosa canina*) using liquid chromatography coupled to electrospray ionisation tandem mass spectrometry and diode-array detection. Rapid Communications in Mass Spectrometry. *16*: 655–662 (2002).

Supplementary Table S3. Pearson correlation matrix among phenolic constituents, total phenolic and flavonoid contents, antioxidant activities, and enzyme inhibitory activities of methanolic extracts from *G. albida*

	PMA	DPPH	ABTS	CUPRAC	FRAP	MCA	AChE	BChE	TYR	AMY	GLU
DPPH	-0.388										
ABTS	-0.219	0.931									
CUPRAC	0.732	-0.184	-0.166								
FRAP	0.558	-0.268	-0.117	0.244							
MCA	-0.136	-0.753	-0.912	-0.097	-0.237						
AChE	-0.872	-0.073	-0.272	-0.687	-0.412	0.568					
BChE	-0.151	0.867	0.897	0.081	-0.409	-0.775	-0.337				
TYR	0.328	-0.883	-0.929	0.156	-0.041	0.873	0.121	-0.764			
AMY	0.716	0.338	0.398	0.713	0.334	-0.617	-0.900	0.464	-0.282		
GLU	-0.820	0.704	0.690	-0.628	-0.469	-0.411	0.455	0.596	-0.729	-0.367	
TFC	0.086	-0.815	-0.714	-0.380	0.200	0.655	0.300	-0.822	0.724	-0.597	-0.363
TPC	0.669	0.099	0.147	0.614	0.790	-0.466	-0.681	0.016	-0.232	0.790	-0.485
Hesperidin	0.582	0.491	0.646	0.522	0.213	-0.823	-0.902	0.689	-0.492	0.914	-0.069
Hyperoside	0.107	0.748	0.779	0.376	-0.312	-0.744	-0.543	0.952	-0.638	0.670	0.336
Luteolin 7-glucoside	0.128	0.776	0.900	0.251	-0.010	-0.944	-0.592	0.910	-0.823	0.660	0.416
Ferulic acid	0.955	-0.212	-0.087	0.623	0.530	-0.218	-0.874	-0.063	0.245	0.795	-0.768
Eriodictyol	0.955	-0.161	-0.018	0.634	0.504	-0.286	-0.914	0.016	0.187	0.825	-0.719
Apigenin	0.935	-0.346	-0.263	0.646	0.673	-0.073	-0.755	-0.280	0.331	0.706	-0.869
Luteolin	0.768	-0.706	-0.675	0.499	0.670	0.366	-0.383	-0.705	0.648	0.294	-0.959
3-Hydroxybenzoic acid	-0.959	0.379	0.291	-0.741	-0.637	0.057	-0.052	-0.778	0.779	0.260	-0.348
4-Hydroxybenzoic acid	-0.929	0.105	0.011	-0.850	-0.512	0.313	0.267	-0.819	0.909	-0.077	-0.089
Apigenin 7-glucoside	-0.159	0.959	0.890	0.083	-0.226	-0.775	-0.912	0.263	-0.290	0.901	-0.810

Protocatechuic acid	-0.828	0.375	0.286	-0.398	-0.777	0.033	-0.266	-0.745	0.608	0.423	-0.332
p-Coumaric acid	0.788	0.252	0.375	0.671	0.479	-0.648	-0.471	0.832	-0.948	0.382	-0.269
Quercetin	-0.548	0.903	0.842	-0.178	-0.542	-0.604	-0.785	-0.207	0.084	0.902	-0.811
Vanillin	0.959	-0.297	-0.164	0.701	0.725	-0.208	0.014	0.838	-0.833	-0.188	0.207
Caffeic acid	0.750	0.273	0.353	0.767	0.411	-0.610	-0.576	0.834	-0.913	0.413	-0.275
Chlorogenic acid	0.095	0.865	0.891	0.251	-0.099	-0.876	-0.877	0.382	-0.547	0.922	-0.777
Syringic acid	0.266	-0.840	-0.879	0.027	0.497	0.719	0.752	0.136	0.221	-0.985	0.753
Taxifolin	0.872	-0.021	0.050	0.646	0.617	-0.350	-0.203	0.875	-0.846	0.020	0.041
Rosmarinic acid	-0.374	0.884	0.843	0.026	-0.494	-0.662	-0.869	-0.078	-0.097	0.952	-0.796
Sinapic acid	0.398	-0.695	-0.692	-0.108	0.396	0.571	0.786	0.095	0.012	-0.808	0.760
Verbascoside	-0.458	0.886	0.860	-0.161	-0.581	-0.629	-0.751	-0.235	-0.017	0.947	-0.765

PMA, phosphomolybdenum assay; DPPH, 2,2-diphenyl-1-picrylhydrazyl radical scavenging activity; ABTS, 2,2′-azinobis-(3-ethylbenzothiazoline-6-sulfonic acid) radical cation scavenging activity; CUPRAC, cupric ion reducing antioxidant capacity; FRAP, ferric reducing antioxidant power; MCA, metal chelating activity; AChE, acetylcholinesterase inhibitory activity; BChE, butyrylcholinesterase inhibitory activity; TYR, tyrosinase inhibitory activity; AMY, α-amylase inhibitory activity; GLU, α-glucosidase inhibitory activity; TPC, total phenolic content; TFC, total flavonoid content.
